# Progress in the study of the effects of selective serotonin reuptake inhibitors (SSRIs) on the reproductive system

**DOI:** 10.3389/fphar.2025.1567863

**Published:** 2025-05-01

**Authors:** Yu Feng, Xiaoyan Qu, Hua Hao

**Affiliations:** ^1^ Department of Pathology, Jingmen People’s Hospital, Jingchu University of Technology Affiliated Jingmen People’s Hospital, Jingmen, China; ^2^ Department of Obstetrics and Gynecology, Yangpu Hospital, School of Medicine, Tongji University, Shanghai, China; ^3^ Department of Pathology, Yangpu Hospital, School of Medicine, Tongji University, Shanghai, China

**Keywords:** SSRIs, reproductive cytogenesis, germ cells, embryonic development, reproductive maturation

## Abstract

In recent years, the increasing number of infertility cases has led to a greater research focus on the reproductive toxicity of drugs due to the fact that some classes of pharmacotherapeutic agents have been found to exert deleterious effects on the reproductive system. Depressive disorders are a class of common mental illnesses that seriously damage human health. The variety of antidepressant drugs is large and the incidence of adverse effects is high. Selective serotonin reuptake inhibitors (SSRIs), as the first-line drugs for the treatment of depression, have remarkable efficacy, but at the same time there is a widespread abuse of them, which not only creates an unfavorable impact on one’s own reproductive system, but also may cause reproductive damage to other non-target populations through pathways, such as the water column. The review provides an introduction to the reproductive toxicity of SSRIs from the aspects of male and female germ cell genesis, embryonic development, reproductive system maturation, and environmental contamination, and it briefly describes the potential mechanisms underlying SSRI-induced reproductive toxicity.

## 1 Introduction

Selective serotonin reuptake inhibitors (SSRIs) increase extracellular serotonin levels by inhibiting the neurotransmitter’s reuptake by synaptic cells, thereby increasing the binding and activation of postsynaptic receptors. The main SSRIs prescribed for clinical use include fluoxetine, paroxetine, sertraline, and citalopram, among others. While SSRIs are efficacious, their safety profile in terms of reproductive system toxicity remains controversial, especially in developing adolescents, fertile adults, and perinatal pregnant women (Dagher et al., 2025). Therefore, the purpose of this review was to summarize the effects of SSRIs on the cytogenesis of male and female germ cells, embryonic development, reproductive system maturation, environmental contamination, to briefly describe the potential mechanisms underlying SSRI-induced reproductive toxicity.

## 2 Effects of SSRIs on reproductive cytogenesis

### 2.1 Effects of SSRIs on male germ cells

**FIGURE 1:**
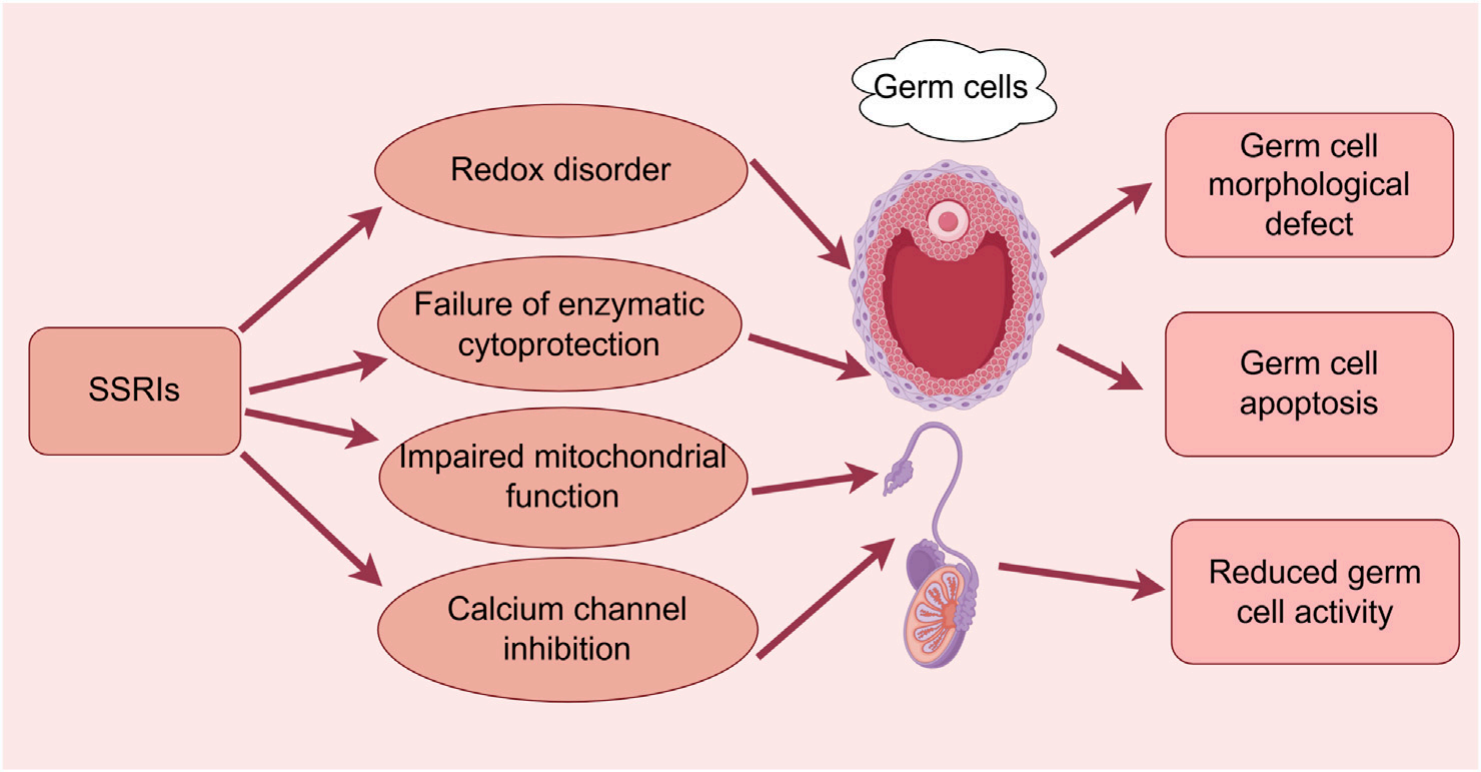
Effects of SSRIs on reproductive cytogenesis. (By Figdraw). SSRIs cause germ cell morphological defects, apoptosis, and reduced activity through redox disruption, enzymatic cytoprotection failure, impaired mitochondrial function, and calcium channel inhibition pathways.

Several studies have found that fluoxetine disrupts the hypothalamus–lobe–testis axis, significantly decreasing the serum concentrations of luteinizing hormone (LH), follicle stimulating hormone (FSH), progesterone, and testosterone, while also affecting several sperm quality parameters, as evidenced by decreased sperm concentrations and viability, increased deoxyribonucleic acid (DNA) fragmentation, and lower reproductive organ weights (Ayala et al., 2018; Beeder and Samplaski, 2020; Monteiro Filho et al., 2014). For example, Câmara et al. (2019) found that short-term fluoxetine treatment resulted in decreased testicular weights, structural disorganization of the seminiferous tubules, and increased germ cell loss and apoptosis in adult rats (Figure 1), which could have been attributed to diminished androgen levels. Fluoxetine also decreased the activity of 17-beta-hydroxysteroid dehydrogenase type 6 (17β-HSD6), a steroidogenesis-related enzyme, and lowered serum testosterone levels by affecting the steroidogenic capacity of Leydig cells; this, in turn, affected the function of androgen-dependent Sertoli cells and perimyocardial myoblast-like cells, ultimately leading to impaired spermatogenesis. Fluoxetine-induced androgen deficiency may lead to the increased expression of ubiquitin carboxyl-terminal hydrolase L1 (UCHL1), which activates the pro-apoptotic factor p53 through deubiquitination and triggers germ cell apoptosis. Based on the aforementioned studies, fluoxetine may negatively affect male reproductive function, especially among androgen-deficient patients who are undergoing fluoxetine treatment for depression; therefore, fertility should be closely monitored in vulnerable populations. However, the sample size in that study was small, as only 16 rats (eight per group) were used in their experiment, and the study duration was short (11 days), which may have affected the statistical significance and generalizability of the results. Although it has been hypothesized that fluoxetine may interfere with spermatogenesis by affecting androgen levels, the concentrations of gonadotropins such as FSH and LH levels were not quantified in the study, and the underlying mechanism of action could not be directly confirmed. Furthermore, fertility testing of fluoxetine-treated male rats was not performed to directly assess its effects on reproductive function, and the study only employed animal models and did not explore the relevance of the findings for human patients. However, Santos et al. (2025) found that at low concentrations, fluoxetine consistently reduced sperm motility while promoting fluctuations in cellular reactive oxygen species (ROS) levels and sperm capacitation, without affecting sperm viability, mitochondrial membrane potential, the acrosome reaction, or chromatin/DNA integrity as shown in Figure 1.

Milosavljević et al. (2022) determined that the negative effects of SSRIs on male fertility were both dose- and treatment-dependent, with the greatest impact observed at high doses and following prolonged exposure, although the effects were reversible after discontinuation (Figure 1). Beeder and Samplaski (2020) also concluded that the effects of fluoxetine appeared to be reversible after discontinuation; however, they observed only minor negative effects at therapeutic doses. Some possible explanations for the contradictory finding include the small sample sizes of the studies, the lack of multicenter or large-sample size studies to verify the reproducibility and generalizability of the results, and the effect of discontinuation conditions and depression itself on the validation of the conclusions of the Chengdu-based study (Figure 1).

Solek et al. (2021a) demonstrated that several antidepressant medications exerted a variety of cytotoxic effects on the spermatogenesis pathway in a time- and concentration-dependent manner (escitalopram, 30 μM; fluoxetine, 5 μM) (Figure 1). The potential molecular mechanisms underlying these effects included disturbances in redox homeostasis (as evidence by the enhanced production of ROS and reactive nitrogen species), failure of enzymatic and nonenzymatic cytoprotective mechanisms involving the glutathione system as well as nuclear factor kappa B (NF-κB) and fibroblast growth factor 2-mediated pathways, and impaired mitochondrial function. In addition, these workers found that antidepressant treatment may lead to defective spindle assembly and altered organelle distribution, with changes in the protein concentrations of small C-terminal domain phosphatases-1 and -3, nuclear mitotic apparatus (NuMa), and calnexin occurring during cell division *in vitro*. The investigation also found that escitalopram and fluoxetine (hydrochloride) exerted cytotoxic effects on mouse spermatogenic cells, leading to micronucleus formation; this, in turn, resulted in the upregulated expression of telomere repeat-binding factor 1/2 (TRF1/TRF2) proteins and strict dependence on p53/p21 signaling, followed by irreversible G2/M cell cycle arrest, ultimately leading to apoptosis. Thus, antidepressants may promote reproductive telomere-centered DNA damage in cell lines (Solek et al., 2021b). However, the study used only two germ cell types (type B spermatogonia and spermatocytes), which made it impossible to evaluate the changes in all cell types during spermatogenesis, and the high concentrations of the drug used in the experiment may have led to exaggerated cytotoxic effects that may not reflect the clinical reality.

Elsedawi et al. (2021) demonstrated that fluoxetine was capable of decreasing serum testosterone levels as well as the height and diameter of the varicocele epithelium, increasing collagen fiber deposition in testicular tissue, and inducing a positive immune response to B-cell lymphoma-2 associated X protein. These fluoxetine-induced changes were attenuated when curcumin was administered in combination with fluoxetine; thus, curcumin may exert protective effects and help attenuate the adverse effects of fluoxetine on the testes. However, the duration of the experiment was just 8 weeks, which made it impossible to determine the long-term consequences of these effects in terms of permanent reproductive dysfunction or recovery after a longer period of time. Additional studies have focused on histopathologic and morphometric changes, with more limited exploration of the mechanisms of action of fluoxetine and curcumin. Although the above studies showed the adverse effects of fluoxetine on male germ cells, the interpretation of the findings is complicated by their limitations, such as their relatively small sample size, the short experimental period, and the inadequate exploration of the mechanism of action; therefore, the findings must be confirmed by more in-depth studies.

Atli et al. (2017) found that sperm concentration, viability, and morphology were affected in male rats after 28 consecutive days of oral administration of sertraline at different doses. Abnormal sperm morphology and an increase in malondialdehyde (MDA) levels were observed in the 10 mg/kg treatment group, whereas the changes were more pronounced in the 20 mg/kg treatment group, which exhibited a decrease in sperm counts, abnormal sperm morphology, DNA damage, degradation of cellular tubular structures, and elevated serum LH and testosterone levels. These changes were accompanied by a decrease in glutathione and an increase in MDA levels, which were indicative of enhanced oxidative stress (OS) (Figure 1). Thus, sertraline induces testicular toxicity in a dose-dependent manner, and enhanced OS and changes in hormone levels may be the key mechanisms. Santos et al. (2025) reached the same conclusion, demonstrating that sertraline had almost no effect at low doses but affected virtually all of the tested parameters at supratherapeutic concentrations. In another study, oral administration of sertraline was found to cause testicular DNA damage and acute testicular injury in male albino rats, as evidenced by significant degeneration and necrosis of germ cells within the seminiferous tubules, as well as interstitial edema and vascular congestion. Sertraline administration increased serum testosterone concentrations and significantly increased lipid peroxidation levels in testicular tissues, whereas glutathione content and catalase activity decreased. Furthermore, wheat germ oil has been shown to significantly reduce lipid peroxidation levels in testicular tissues and improve antioxidant defenses to protect against sertraline-induced testicular damage (Hamdi, 2019). However, indicators such as OS and DNA damage provide no information regarding the specific molecular mechanisms underlying the protective effects of wheat germ oil, and few studies have investigated the dose-effect relationship to determine the optimal therapeutic dose and assess the safety profile.

Sertraline was shown to impair both the ligand-induced acrosome reaction and spermatozoa entry into viscous media and it almost completely inhibited the sperm-specific Ca^2+^-permeable ion channel (CatSper), with a significant inhibitory effect on prostaglandin-induced Ca^2+^ influx (Rahban et al., 2021). These findings imply that the off-target effect of sertraline on CatSper in human spermatozoa may affect fertility (Figure 1). The above studies demonstrated the adverse effects of sertraline on male germ cells; however, there were important limitations to consider, including small sample sizes, a lack of long-term observation, the use of a single species or strain, the lack of in-depth exploration of cellular or molecular mechanisms, failure to assess other aspects of reproductive function, a lack of validation of the clinical relevance, and a failure to consider individual differences in drug dosages. Thus, the conclusions of those studies must be further validated, and a wider range of studies is necessary to ensure the reliability of the findings.

Ilgin et al. (2017) administered citalopram hydrobromide orally to rats at doses of 5, 10, and 20 mg/kg/day for 28 days. Sperm concentrations, viability, and morphology were measured using a computer-assisted sperm analysis system, and sperm DNA damage was detected using the comet assay. It was found that citalopram hydrobromide treatment resulted in decreased sperm concentrations, abnormal sperm morphology, and increased sperm DNA damage. In terms of hormone concentrations, LH levels were elevated at all three dosages, testosterone levels were elevated in the 5 and 10 mg/kg groups, and OS was enhanced in the 10 and 20 mg/kg groups. Therefore, it was concluded that citalopram hydrobromide induced testicular damage in male rats as well as OS and hormonal changes, which are considered to be an important cause of reproductive disorders. In studies conducted by Solek et al. (Solek et al., 2021a; Solek et al., 2021b), animal experiments were performed in mice to elucidate the potential mechanisms underlying the cytotoxic effects of escitalopram on spermatogonial cells, similar to those of fluoxetine (see above). In addition, randomized controlled trials conducted by Moradi et al. (Moradi et al., 2023; Moradi et al., 2024) demonstrated that melatonin and vitamin C restored spermatogenesis by improving sperm counts, motility, viability, morphology, and chromatin integrity, and testosterone levels and testicular histopathology were significantly improved in the melatonin and vitamin C treatment groups (Figure 1). In addition, melatonin and vitamin C restored spermatogenesis by increasing the total antioxidant capacity (TAC), and decreasing nitric oxide (NO) and testicular MDA levels restored the antioxidant status. More notably, citalopram treatment significantly increased the number of TdT-mediated dUTP nick end labeling (TUNEL)-positive cells, whereas melatonin and vitamin C treatment significantly attenuated citalopram-induced apoptosis. They concluded that melatonin and vitamin C treatment protected against citalopram-induced testicular injury by modulating nitrooxidative stress and apoptosis (Figure 1). Collectively, the aforementioned animal studies have shown that citalopram damages male germ cells and alters related mechanisms. Although protective drugs have also been discovered, the studies had limitations, including their small sample sizes and failure to explore the mechanisms in-depth, and the clinical relevance of the findings has yet to be verified; thus, more objective evidence is needed to support their conclusions.

### 2.2 Effects of SSRIs on female germ cells

Romero-Reyes et al. (2016) found that acute fluoxetine administration in prepubertal female rats resulted in increased estradiol concentrations, whereas subchronic administration led to increased serotonin concentrations in the ovaries, a decrease in the number of ovulations, a greater number of atretic follicles, and oocyte fragmentation. These results suggest that fluoxetine may affect follicular development and the ovulatory process by acting on the ovaries or the hypothalamic–pituitary axis. Moore et al. (2015) found that perinatal fluoxetine exposure may result in irregular reproductive cycles in adult offspring, as evidenced by a prolonged estrous period, increased numbers of secondary and total follicles, and enhanced apoptosis in ovarian cells. In addition, altered expression of genes that regulate serotonin signaling and function in the ovaries of the offspring of fluoxetine-treated animals may occur in combination with the increased expression of components of the regulatory loop of the core circadian locomotor output cycle Kaput (*Clock*) gene, suggesting interactions between serotonin and *Clock* gene signaling pathways can lead to an altered reproductive phenotype.

Milosavljević et al. (2019) demonstrated that SSRIs affect voltage-gated Na^+^, K^+^, and Ca^2+^ channels in smooth muscle cells, with the likelihood of altered tubal peristalsis varying in accordance with the drug being administered (Figure 1). In that study, escitalopram and paroxetine produced a concentration-dependent increase in spontaneous contractions in isolated fallopian tubes; the significant stimulatory effects had the potential to interfere with tubal function, which could, in turn, affect fertility. However, the limited sample size of their study (only 20 patients) may have limited the generalizability of the results, and they failed to investigate the specific receptor-mediated mechanism underlying the effect of SSRIs on tubal motility and the fallopian tubes at different stages of the menstrual cycle. Based on the fact that contractile activity of the fallopian tubes is critical for sperm transport, egg fertilization, and early embryonic development, caution must be exercised when SSRIs are clinically recommended in women of childbearing age. In a prospective cohort study involving a clinical population of 1,228 women of childbearing age who were planning to conceive naturally, those who were exposed to SSRIs experienced a 24% reduction in the ability to conceive, with fluoxetine exerting an even more pronounced effect than other SSRIs, as well as a 9% reduction in the incidence of live births; therefore, SSRIs in general and fluoxetine in particular should be used with caution (Sjaarda et al., 2020).

Observational studies support the use of SSRIs during *in vitro* fertilization (IVF), as they are effective in relieving symptoms of depression/anxiety and do not have a significant negative impact on IVF outcomes. In terms of natural conception, the effect of SSRIs on female fertility remains unclear; however, certain studies have suggested that they may reduce the probability of conception. Overall, it is recommended that SSRIs be avoided in male patients of childbearing age, whereas their use during IVF appears to be reasonable in women with depression or anxiety disorders (Milosavljević et al., 2022). Most of the studies conducted to date have been observational in nature, and there is a lack of randomized controlled trials; furthermore, the number of studies that met the inclusion criteria was small, depression and anxiety themselves may affect fertility, and there is significant heterogeneity in the literature, especially in the measurement of fertility parameters. The assessment of fertility in women is more complex than in men, and there is a lack of direct biomarkers. More studies that address these limitations are required to confirm the effects of SSRIs on natural conception.

## 3 Effects of SSRIs on embryonic development

**FIGURE 2:**
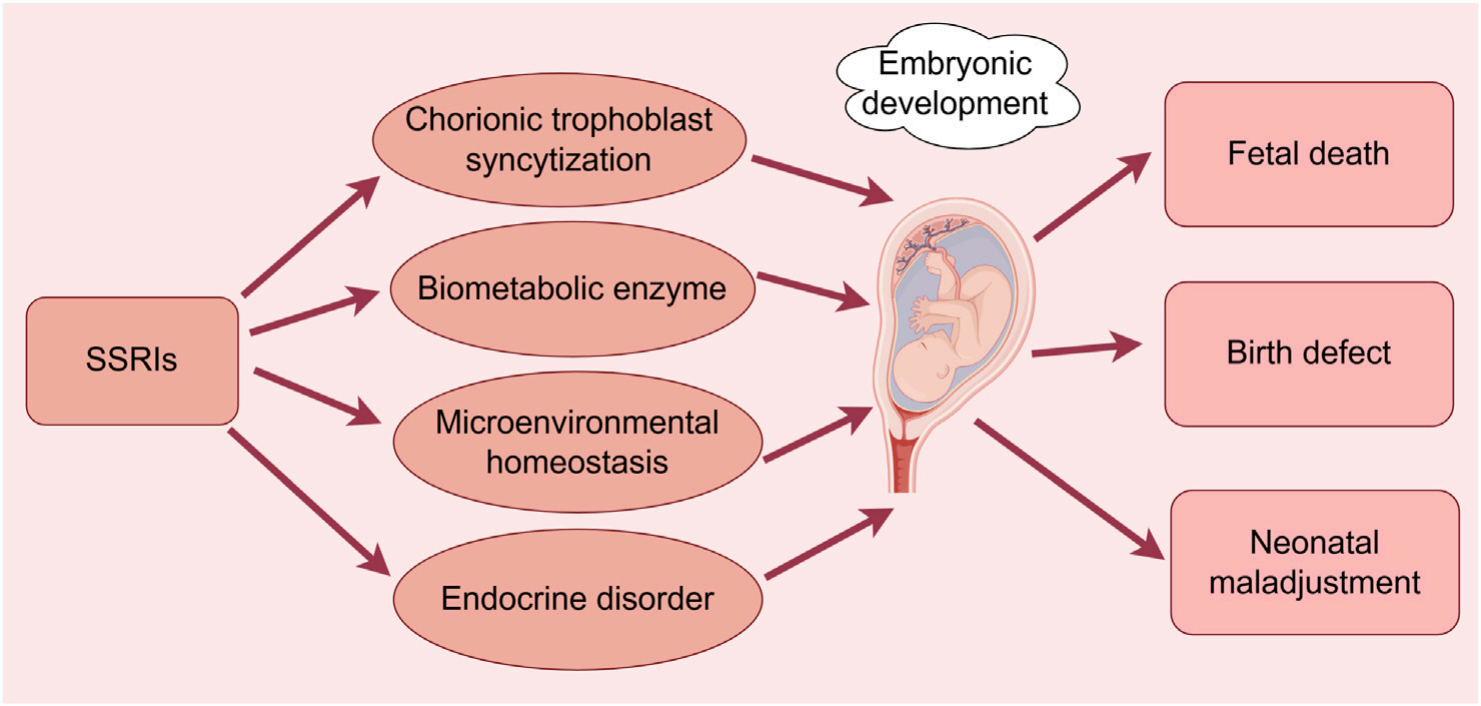
Effects of SSRIs on embryonic development. (By Figdraw). SSRIs cause fetal death, congenital malformations, and neonatal maladaptation through chorionic trophoblast syncytization, biometabolic enzymes, disruption of microenvironmental homeostasis, and endocrine disruption.

Exposure to any SSRIs such as citalopram, paroxetine, and sertraline at any time during pregnancy has been shown to increase the risk of fetal mortality (Figure 2) (Desaunay et al., 2024; Domingues et al., 2022a). In addition, the use of SSRIs during pregnancy increases the risk of congenital malformations, congenital heart defects, cleft palate malformations, preterm delivery, lower fetal weight, increased visceral and skeletal abnormalities, neonatal adaptation symptoms, and persistent pulmonary hypertension in newborns. Maternal toxicity induced by SSRIs like sertraline has been shown to manifest as a significant reduction in weight gain during pregnancy, and the risk of preeclampsia and *postpartum* hemorrhage may also be increased; therefore, it is recommended that the lowest effective dose be used for the treatment of depressive or anxiety disorders to ensure therapeutic benefits in the mother while simultaneously reducing fetal risk (Desaunay et al., 2023; Bandoli et al., 2020; Cabrera et al., 2020; Palmsten et al., 2020).

An umbrella review of meta-analyses of observational studies archived in the PubMed and Embase databases that included 1,175,720 women who had used antidepressants during pregnancy reported that the use of SSRIs during pregnancy was associated with a 43% increase in the risk of preterm labor, a 43% increased risk of respiratory distress, a 33% associated with respiratory distress, a 26% increased risk of cardiovascular malformations, and a decrease in 1-min Apgar scores (Biffi et al., 2020). A meta-analysis conducted by Vlenterie et al. (2021) based on individual participant data also found that depressive symptoms experienced during pregnancy or clinically diagnosed depression were associated with preterm labor and low Apgar scores, and the use of SSRIs may independently increase the risk of preterm labor and the likelihood of achieving low Apgar scores. However, they determined that the strength of the evidence for these associations was limited, and some of the studies did not adequately correct for potential confounders such as the severity of depression, hormone levels, and lifestyle factors, among other variables. These issues may have biased the results, and more prospective studies and large-scale collaborations are needed to standardize the reporting of analytical results to facilitate a more accurate assessment of the risks associated with antidepressant use during pregnancy. In another cohort study based on 280,090 term infants born at Kaiser Permanente hospitals in northern California between 2011 and 2019, 7,573 (2.7%) infants that had been exposed to SSRIs in late pregnancy, and 11.2% experienced delayed adaptation (defined as a 5-min Apgar score ≤5, resuscitation at birth, or the administration of respiratory support owing to admission to the neonatal intensive care unit), compared with 4.4% in the unexposed group; after correction, the association between SSRI exposure and delayed adaptation remained significant. The association between SSRI exposure and delayed adaptation was dose-dependent, with higher equivalent doses of sertraline being associated with a greater risk, and escitalopram and fluoxetine use was associated with the highest risk of delayed adaptation. However, no significant associations were observed between SSRI exposure and pulmonary hypertension, hypoxic–ischemic encephalopathy, or convulsions (Cornet et al., 2024). A systematic review and meta-analysis based on a total of 16 studies archived in the PubMed and Embase databases that assessed pregnancy outcomes in 4,564,798 women revealed that mothers who used SSRIs had an odds ratio (OR) of 1.22 (95% confidence interval (CI): 1.12–1.33) for congenital heart disease (CHD) in their fetuses, with an increased risk of approximately 22% and an OR of 1.53 (95% CI: 1.25–1.88) for paroxetine, an increased risk of approximately 53% and an OR of 1.28 (95% CI: 1.01–1.62) for fluoxetine, and an increased risk of approximately 28% and an OR of 1.28 (95% CI: 1.14–1.45) (De Vries et al., 2021).

Gao et al. (2018) systematically reviewed the association between SSRI use in early pregnancy and the risk of fetal congenital malformations by searching for relevant studies in the PubMed, Embase, Web of Science, and Cochrane Library databases; ultimately, 29 cohort studies involving more than 9 million deliveries were identified. These investigations described that SSRI use in general was associated with an increased risk of major congenital anomalies (MCAs) and CHD in the fetus, that sertraline use in early pregnancy was associated with an increased risk of congenital malformations such as ventricular septal defects and respiratory defects, and that the use of citalopram, fluoxetine, and paroxetine was associated with an increased risk of both MCAs and CHD. However, when the analysis was restricted to women with a psychiatric diagnosis, there was no significant change in risk. They suggested that SSRIs are associated with an increased risk of fetal congenital malformations, although this risk is small and does not support the possibility of a significant teratogenic effect of SSRIs. While caution should be exercised when using SSRIs during pregnancy, discontinuing treatment may be more harmful to mothers and infants with major depressive disorder. A case-control study based on the National Birth Defects Prevention Study (NBDPS) that included data from 30,630 mothers of infants with birth defects and 11,478 control mothers with infants without major birth defects from the period of October 1997 to December 2011 found that the use of fluoxetine and paroxetine was associated with certain CHDs. Although this association was attenuated after partial correction for underlying disease, the associations with other non-cardiac defects such as diaphragmatic hernia persisted (Anderson et al., 2020). However, that study was based on self-reported medication use assessed *via* telephone interviews without direct access to medical records or diagnostic information, which may have affected the accuracy of the data. A systematic review and meta-analysis conducted by Masarwa et al. (2019) indicated that exposure to SSRIs during pregnancy was associated with an increased risk of persistent pulmonary hypertension (PPHN), with a higher risk observed in the later stages of pregnancy (>20 weeks). A network meta-analysis showed that sertraline may be associated with the lowest risk of PPHN in comparison with that of all other SSRIs, suggesting that sertraline may have the best safety profile.

Kildegaard et al. (2024) followed all Danish singleton children born between 1997 and 2015 over a period of 15 years and found that children exposed to SSRIs prenatally had an overall increased risk of childhood disorders of gut–brain interactions (DGBI) (relative risk: 1.08), with the main driver being functional constipation (relative risk: 1.19), suggesting that prenatal SSRI exposure is associated with an increased risk of developing functional constipation. These findings are consistent with a large body of preclinical data supporting the critical role of serotonin in modulating gut development and function, although further research is necessary to determine the long-term effects of maternal depression and SSRI exposure on the development of common gastrointestinal disorders.

Sestario et al. (2022) found similar embryonic effects and fetal malformations when escitalopram was administered in mice during pregnancy, including changes in the number of reinstatements, post-implantation loss, reduced fetal survival, reduced kidney size, supra-occipital ossification insufficiency, and the absence of ribs and phalanges. Fricke et al. (2023) demonstrated that low-dose lactational fluoxetine exposure caused more damage to the bones of the offspring than either fetal and postnatal developmental exposure to low-dose fluoxetine or lactational exposure to high-dose fluoxetine. Furthermore, developmental fluoxetine exposure in general and lactational fluoxetine exposure in particular exerted long-lasting effects on the bone and body composition of adult mice.

Dagher et al. (2025) reviewed the clinical safety of antidepressant medications and their relationship to maternal depression and neonatal outcomes to compare the therapeutic effects of antidepressants with the deleterious effects induced by a lack of treatment in mothers and children, which has been well-researched. Overall, they concluded that the effects of SSRIs on fetal health and their adverse effects on childhood development were limited, although individuals should be counseled about the risks and benefits of antidepressant treatment before and during pregnancy, as the refusal of treatment may result in negative outcomes. In addition, newer treatments, such as ketamine and κ-opioid receptor antagonists, warrant further study to verify their effects during pregnancy. The safety of antidepressant use during pregnancy remains controversial due to an incomplete understanding of the means by which drug exposure affects fetal development, brain maturation, and offspring behavior. Pregnant individuals are particularly vulnerable to the effects of antidepressants, as pregnancy is a highly stressful experience for many women, and stress is recognized as the predominant risk factor associated with mood or anxiety disorders; therefore, many factors must be considered, and treatment should be individualized.

In summary, some controversies remain regarding the effects of SSRIs on embryonic development, and heterogeneity may exist owing to the different definitions of congenital malformations used in different studies, especially those related to CHD (Figure 2). The fact that most of the studies included only live-born infants and did not record or observe stillbirths, spontaneous abortions, or induced abortions due to severe malformations, potentially led to underestimations of the strength of the associations between SSRIs and fetal congenital malformations. More rigorous basic research studies and clinical trials are needed to verify these effects.

Studies investigating the potential mechanisms underlying the effects of SSRIs on embryonic development are ongoing. The effects of SSRIs on mortality in young rats may be mediated by SSRI-induced placental insufficiency rather than direct toxic effects on neonatal development and mortality (Domingues et al., 2022a). Sahlman et al. (2022) investigated the effects of SSRIs such as escitalopram in women who used them during pregnancy; they observed that the mean enzymatic activities of xenobiotic metabolizing enzymes such as cytochrome P450 1A1 (CYP1A1), aromatase (CYP19A1), and glutathione-S-transferase (GST) were lower in three placental microsomes and subcellular membrane fractions. After adjusting for other factors, significant differences in the enzymatic activities of CYP19A1 (*p* = 0.001) and CYP1A1 (*p* = 0.002) were still observed between the study groups, although there was no significant effect on placental GST activity. The findings suggest that SSRIs alter the activities of placental enzymes, which could lead to disturbances in maternal steroid hormone homeostasis and biometabolism, thereby posing a risk to both the developing fetus and the pregnant woman. Clabault et al. (Clabault et al., 2018a) found that fluoxetine and sertraline administered at therapeutic doses significantly reduced the activity of extraembryonic trophoblastic cells (evTBs) when JEG-3 cells and HIPEC cells were used as the evTBs model. At a concentration of 10 μM, the SSRIs reduced the cell proliferation rate by 94% and 100%, respectively, for JEG-3 cells and by 58.6% and 100%, respectively, for HIPEC cells. In addition, norfloxacin was shown to increase the activity of metalloproteinase 9 (MMP-9) when administered at various concentrations, whereas sertraline significantly increased the messenger ribonucleic acid (mRNA) levels of matrix metalloproteinase inhibitor-1 (TIMP-1) and disintegrin and metalloproteinase 10 (ADAM-10) at different concentrations. Thus, SSRIs may affect the homeostasis of evTBs when administered at therapeutic levels. The same study also demonstrated that neither fluoxetine nor its metabolites norfluoxetine and sertraline affected the proliferation and viability of human chorionic tumor cells (BeWo cells) or the apoptosis of primary trophoblasts. In a concentration-dependent manner, sertraline exposure increased the fusion of primary chorionic trophoblast cells without affecting the fusion of BeWo cells. Sertraline also affected the secretion of human chorionic gonadotropin β (β-hCG) by BeWo cells, whereas none of the SSRIs affected β-hCG secretion by primary trophoblasts. In addition, norfloxacin exposure increased the expression levels of the genes encoding chorionic gonadotropin beta (CGB) and gap junction protein alpha 1 (GJA1), both of which are biomarkers of syncytization, in BeWo cells, whereas in primary trophoblasts, none of the SSRIs tested affected the expression of these genes. These findings suggest that SSRIs affect the syncytization of chorionic trophoblast cells in a structure- and concentration-dependent manner, implying that certain SSRIs may impair placental health (Clabault et al., 2018b). Collectively, these studies revealed that SSRIs may interfere with placental function by affecting the proliferative, migratory, and invasive capacities of evTBs and the syncytization of chorionic trophoblast cells in the placenta while also emphasizing the importance of using primary cells when investigating the effects of drugs on placental function. However, the study did not explore the long-term effects of SSRIs on placental function and lacked validation through *in vivo* models. Future studies must further explore the specific mechanisms by which SSRIs affect placental function, and the findings must be validated using *in vivo* models. Furthermore, studies should consider the effects of drug metabolism, the placental barrier, and fetal sex to more fully assess the safety of SSRI use during pregnancy.

In terms of cardiovascular effects, Arumugasaamy et al. (Arumugasaamy et al., 2019) found that both fluoxetine and sertraline slowed calcium oscillations in cardiomyocytes, inducing the increased secretion of troponin T and creatine kinase-MB, while also decreasing the secretion of N-terminal prohormone of brain natriuretic peptide (NT-proBNP), all three of which are key biomarkers of cardiac injury. Changes in calcium oscillations in cardiomyocytes were further amplified following indirect exposure to SSRIs through the placental barrier, suggesting that the placental barrier may influence the effects of drugs like SSRIs on cardiomyocytes. However, the specific mechanisms underlying the changes in calcium oscillations and biomarker levels observed in the study are unclear, and the effects of SSRIs on long-term cardiomyocyte functions, such as contractile activity or electrophysiological properties, have not been assessed. Warkus et al. (Warkus and Marikawa, 2018) demonstrated that neither fluoxetine nor its main metabolite norfluoxetine (norfluoxin) had an effect on cardiomyocytes in the P19C5 embryoid body (EB) model in mice. More specifically, norfluoxetine significantly altered the morphology of EBs at concentrations of 6 mM and above, as evidenced by a reduction in the EB area and a decrease in axial elongation. Fluoxetine also significantly altered the expression pattern of several key developmentally regulated genes, especially those related to mesodermal differentiation, such as brachyury, *Mixl1*, and *Cdx1*. Fluoxetine affects embryonic morphogenesis by inhibiting the activity of the Wnt signaling pathway and reducing cell proliferation, and activation of the Wnt signaling pathway partially mitigates the adverse effects of fluoxetine on embryonic morphology through a mechanism of action that is not directly related to the inhibition of serotonin reuptake in the synapse. These findings provide new insights regarding the safety of fluoxetine use during pregnancy and suggest that they may affect embryonic development through an unconventional mechanism.

Another study showed that fluoxetine treatment in ewes during the last month of pregnancy reduced placental nodule growth, shortened the gestational period by 4.5 days, and decreased the birth weight of lambs following exposure *in utero*. After birth, the lambs exhibited metabolic acidosis, a blood disorder involving changes such as decreased blood pH, lower total carbon dioxide, bicarbonate, and ionized calcium levels, and base excess, as well as increased lactate concentrations (Domingues et al., 2022b). In pregnant sheep, fluoxetine treatment has also been shown to decrease prenatal circulating calcium levels, an effect that was transient and disappeared post-treatment; furthermore, fluoxetine treatment resulted in a decrease in estradiol concentrations, whereas the levels of the steroids cortisol and progesterone remained unchanged (Domingues et al., 2023a). In contrast, sertraline alone has been shown to increase prolactin levels and significantly prolong gestation in female rats (Yilmaz et al., 2024). Lyons et al. (Lyons et al., 2016) further observed that SSRIs such as fluoxetine and sertraline inhibit the activity of dopaminergic neurons in neuroendocrine leaky nodules through the activation of G protein-coupled inwardly rectifying K^+^-like currents mediated by the 5-hydroxytryptamine 1A receptor, leading to reduced intrinsic excitability and slower tuberoinfundibular dopamine (TIDA) network rhythms, thereby increasing prolactin secretion.

Another systematic review and comprehensive analysis indicated that antidepressants vary in their ability to enter amniotic fluid, cord blood, and breast milk, with citalopram having the highest permeability (mean permeability of active molecules: 2.03, range: 0.35–6.97) and fluoxetine having the lowest permeability (mean permeability of active molecules: 0.11, range: 0.02–0.20) (Schoretsanitis et al., 2021). Thus, the ability of different SSRIs to enter fetal, neonatal, and infantile bodily fluids can vary considerably, resulting in potentially different effects. Fluoxetine administration to treat depression during pregnancy has been shown to induce greater changes in gene expression in the placenta of male infants than in that of female infants, emphasizing its sex-specific effects (Staal et al., 2024). The aforementioned studies suggest that SSRIs may contribute to the development of fetal myocardial injury, while also affecting maternal calcium homeostasis and steroid metabolism as well as inducing endocrine dysfunction, intrauterine fetal growth restriction, and neonatal maladaptation to the extrauterine environment (Figure 2). The studies also had some shortcomings, as some only administered fluoxetine at the end of pregnancy and could not assess the effects of the drug throughout the entire pregnancy period, some did not explore the specific mechanisms of fluoxetine-induced fetal acidosis, hypocalcemia, and altered maternal estradiol metabolism, and some failed to assess the maternal and fetal health status over a long-term follow-up period. The study was based only on data from the published literature, did not allow for prospective or randomized controlled trials, and did not explore the direct associations between drug penetration ratios and adverse fetal or neonatal outcomes.

## 4 Effects of SSRIs on reproductive maturation

**FIGURE 3:**
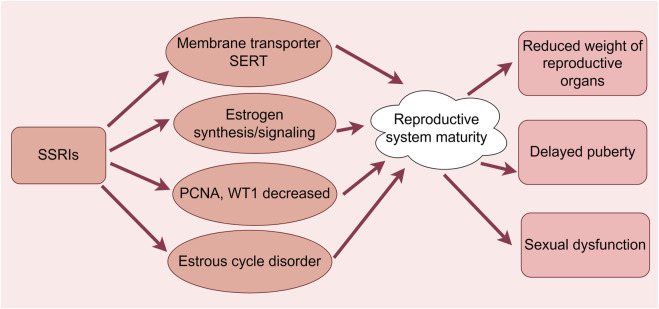
Effects of SSRIs on reproductive maturation. (By Figdraw). SSRIs cause reduced weight of reproductive organs, delayed puberty, and sexual dysfunction through membrane transporter SERT, estrogen synthesis/signaling, reduced PCNA and WT1 and dysregulation of the estrous cycle.

Sertraline-exposed male offspring have been shown to exhibit lower body weights at various stages of growth, as well as lower anal spacing, delayed reflex development, and altered testicular histology; in adulthood, sperm quality was also altered without affecting natural fertility (Figure 3) (Lozano et al., 2023). ElMazoudy et al. (ElMazoudy et al., 2020) examined the reproductive effects of cumulative dosing of sertraline from the juvenile period to puberty in male rats. It was determined that sertraline-treated male rats exhibited a number of abnormal reproductive changes between postnatal day 28 and puberty, including significant decreases in body, liver, kidney, testicular, and epididymal weights. Also noted were significantly lower LH, FSH, testosterone, and 17β-HSD concentrations, diminished sperm counts, viability, and survival (Figure 3). These changes were accompanied by increases in sperm morphological defects, testicular histology revealing the presence of germ cells, and severe degeneration of supporting and mesenchymal cells. Male rats receiving 1.2 mg/kg sertraline cumulatively until postnatal day 126 exhibited significantly reduced male potency (based on various indices of mating and fertility), while cumulative treatment significantly increased the number of fetal congenital malformations and reduced the numbers of implantation sites and surviving fetuses. Thus, sertraline interferes with the function of the hypothalamic–pituitary–testicular and hypothalamic–pituitary–adrenal axes by affecting the wide distribution of serotonin receptors, thereby leading to reduced fertility in male rats (Figure 3).

Munkboel et al. (2018) found that sertraline causes impaired the steroidogenesis of sex hormones in male rats through the direct inhibition of steroid synthesis and its indirect effects on gene expression, and plasma LH levels and LH/testosterone ratios were significantly increased, potentially triggering compensatory hypogonadism. de Oliveira et al. (2013) determined that fluoxetine exposure resulted in suppressed and delayed testicular development; more specifically, exposure to 10 and 20 mg/kg fluoxetine reduced the body and testicular weights in offspring, decreased spermatogonial tubule and epithelial volumes, altered the seminiferous tubule length and the number of supporting cells, and increased the number of seminiferous tubules lacking a lumen. Quiarato Lozano et al. (Quiarato Lozano et al., 2022) demonstrated that the fetal testes of sertraline-exposed rats exhibited a large number of eosinophils, a reduction in the nuclear volume of testicular interstitial cells, a decrease in the expression of proliferating cell nuclear antigen (PCNA) and Wilms tumor protein (WT1), and delayed puberty. However, the limitations of animal models, the lack of long-term follow-up data, single-dose experiments (1.2 mg/kg), and the lack of mechanistic studies limit the generalizability and reliability of the results.

Serotonin is a regulator of oocyte maturation in many animal species, and the accumulation of exogenous maternal serotonin is mediated by the activity of the membrane-bound serotonin transporter (SERT). Ovarian granulosa cells regulate the accumulation of serotonin in oocytes through the uptake and degradation of serotonin by SERT and monoamine oxidase (MAO), respectively, and granulosa cells form a functional barrier that protects oocytes from exogenous serotonin and may play an important role in oocyte maturation and early embryonic development (Alyoshina et al., 2022). Alyoshina et al. (Alyoshina et al., 2023) reported that differences in SERT activity in mammalian oocytes were not associated with follicular markers of apoptosis but were positively correlated with indicators of follicular growth such as granulosa cell proliferation and follicle size. They observed that as oocytes matured, SERT expression and activity increased. Following seven consecutive days of treatment with fluoxetine, the serotonin content in growing germinal vesicle oocytes and ovulation-matured MII oocytes was significantly decreased, and the expression and activity of SERT were reduced, leading to impaired oocyte maturation (Figure 3). Another study observed disruption of the motility cycle in mice treated with high-dose fluoxetine. Although uterine expression levels of estrogen receptor α, G protein-coupled estrogen receptor, and steroid synthase were upregulated in the high-dose group, the decreased expression of connexin 43 and alkaline phosphatase as well as the increased expression of insulin-like growth factor-binding protein three and monoamine oxidase were consistent with decreased estrogen signaling and lower uterine weights. Thus, it was suggested that fluoxetine can regulate estrogen synthesis/signaling and cause dysregulation of the estrous cycle, thereby causing sexual dysfunction (Figure 3) (Domingues et al., 2023b).

Marshall et al. (2020) found that fluoxetine-treated rats spent significantly less time and were significantly less active than either stimulus animal in a partner preference test and exhibited a significantly longer contact to return latency to ejaculation and spent less time with other males during rhythmic mating. Thus, even with sexual experience, fluoxetine interferes with sexual function. Furthermore, the increased ejaculatory latency induced by SSRIs may result in a number of negative effects on semen quality, including higher DNA fragmentation in sperm, increased prolactin (PRL) levels, decreased testosterone levels, and sexual dysfunction in some men, potentially inhibiting male reproduction (Figure 3) (Drobnis and Nangia, 2017). Despite some negative effects on semen quality in men, fluoxetine has been shown to significantly improve intravaginal ejaculatory latency, ejaculatory control, and psychological distress in patients with primary premature ejaculation; however, continual use exceeding 6 months can lead to poor results (Jenkins et al., 2019). In summary, SSRIs may have detrimental effects on the maturational aspects of the male and female reproductive systems; however, due to limitations in study design and insufficient data, the lack of relevant clinical trials, and limited exploration of the molecular mechanisms, the current findings are insufficient to generate adequate guidelines for use in clinical practice, and more high-quality studies are needed to further clarify the reproductive toxicity of SSRIs.

## 5 Effects of SSRIs on the reproductive system of aquatic organisms

SSRIs are a major class of drugs of environmental concern, and fluoxetine, a psychoactive agent, is one of the best-selling prescription antidepressants in the world, which increases the likelihood of aquatic environmental contamination through municipal, industrial, and hospital discharges, potentially inducing sublethal effects in non-target species.

Henry et al. (2022) found that even at very low concentrations (i.e., ng/L levels), SSRIs alter the behavior of aquatic animals. The study used freshwater snails in a multigenerational exposure experiment and showed that those exposed to fluoxetine exhibited significantly reduced behavioral plasticity and significant behavioral reproducibility in terms of their activity levels compared to those of unexposed snails. In addition, snails exposed to fluoxetine exhibited reduced egg production, and marginal non-significant differences in morphology compared to that of the unexposed group. These results highlight the potentially deleterious effects of chronic fluoxetine exposure on non-target organisms in the environment.

In a study conducted by Bertram et al. (2018), experiments were performed using eastern mosquito fish, which were assigned to a control group or one of two fluoxetine-exposed groups (40 ng/L and 400 ng/L at the low and high concentrations), with an exposure time of 30 days. The number of mating attempts was significantly higher in the male mosquitofish who were exposed to high-concentration fluoxetine in the absence of competition, whereas no significant difference was observed in the presence of competition. Sperm counts were significantly increased in fluoxetine-exposed male mosquitofish, but sperm quality (e.g., motility, vigor, etc.) was not significantly affected. Body condition indices were significantly lower in male mosquitofish exposed to low-concentration fluoxetine, with no significant effect on body length, body weight, and reproductive organ length.

Fluoxetine in the environment may interfere with reproductive processes in wildlife by affecting sexual selection mechanisms. Hong et al. (Hong et al., 2022) exposed zebrafish to citalopram at doses of 0.1–100 μg/L separately throughout their life cycle from the embryonic stage to adulthood. After 180 days of exposure, the lowest observable effective concentrations (LOECs) at which citalopram significantly reduced the mean spawning of adult fish were 10 and 1 μg/L, respectively, whereas the fertilization rate was not significantly altered. In addition, the study found that citalopram did not affect sperm quality, although it did cause a significant reduction in sperm production after exposure to concentrations of 1–10 μg/L Reproductive behaviors such as the duration of mating and mating intervals were reduced in antidepressant-exposed adults. Transcriptomic analysis of the whole ovary revealed that exposure to citalopram throughout the life cycle significantly affected the signal transduction of Na^+^/Cl^−^-dependent neurotransmitter transporters. Immune system-mediated ovarian regeneration and energy metabolism regulated by creatine metabolism were suggested as novel potential mechanisms. The major teratogenic effects induced by fluoxetine in zebrafish embryos included pericardial edema, delayed hatching, spinal changes, and craniofacial malformations. As the concentrations increased, biomarkers of oxidative damage affected the embryos more than antioxidant enzymes did. Due to its high teratological potential, fluoxetine is a dangerous drug to be exposed to in the early life stages of zebrafish, and fluoxetine-induced OS may be involved in this toxicity (Orozco-Hernández et al., 2022). Huang et al. (Huang et al., 2020) used an ultra-low-input RNA sequencing method (SMART-seq v.4 technology) to investigate the effects of two SSRIs (fluoxetine and paroxetine) on zebrafish larvae. In the brain, they observed changes in the expression of genes associated with mitochondrial and neuronal structure, mitochondrial respiration, and neurodevelopmental processes, with key genes (e.g., *fkbp5* and *ucp2*) correlating with the response to SSRIs and neurodevelopment. Furthermore, they observed that SSRI exposure may lead to neurodevelopmental abnormalities as a result of mitochondrial dysfunction. Studies have shown that paroxetine and other SSRIs have been discovered in the tissues of invertebrates and fish; however, there is a complete lack of environmental monitoring of these drugs.

The primary purpose of the National Institute for Occupational Safety and Health (NIOSH) Hazardous Drug List is to provide information to healthcare professionals to ensure appropriate protective measures are being employed to prevent exposure to drugs and other chemicals on the job. However, the list also provides useful information for other professionals such as environmental scientists and researchers. The paucity of relevant environmental data for certain hazardous pharmaceuticals must be addressed to help prioritize compounds that require further research (Abajo et al., 2023).

In summary, SSRIs are associated with the potential risk of pharmaceutical contamination in aquatic environments that may impact the reproductive development of aquatic animals, with implications for the adaptation of populations in response to current and future environmental challenges. However, further cross-species studies, long-term ecological monitoring, and in-depth mechanistic explorations are needed in the future to overcome the limitations of current studies. Exposure to human health hazards through direct contact or the food chain must also be considered in order to comprehensively assess the impact of SSRI contamination on ecosystems and to develop relevant regulatory policies to minimize the environmental impact of pharmaceutical agents.

## 6 Limitations and future directions

The above studies discussed the effects of SSRIs in the reproductive system, but the following limitations exist: the small sample sizes of several studies and the lack of large-scale, multicenter, randomized controlled trials; the effects of a variety of potentially confounding factors, such as the severity of depression, the use of other medications, and other underlying disorders; most of the existing studies are observational or animal experiments, and it is difficult to extrapolate them directly to humans; the toxic effects of SSRIs on the mechanism of toxic effects of SSRIs on the reproductive system has not been fully clarified, and although some studies have explored mechanisms such as oxidative stress, changes in hormone levels, and apoptosis, they lack systematic and in-depth nature, and lack *in vivo* model validation. Insufficient research on the differences in the effects of SSRIs in different genders and developmental stages (e.g., embryonic, puberty, adulthood); research focuses on the effects of short-term exposures or low-concentration exposures on aquatic organisms, and long-term ecological monitoring of SSRIs is rarely performed, and there have been few cross-species studies that have investigated the effects of short-term or low-concentration exposure on aquatic organisms. Even fewer studies have investigated the effects of SSRI metabolites after degradation in the ecological and their potential reproductive toxicity.

In terms of future research directions, large-scale, randomized, placebo-controlled clinical trials must be conducted that fully consider the effects of potential confounding factors to clarify the long-term effects of SSRIs on male and female fertility, to assess the recovery of reproductive function after drug discontinuation, and to clarify their reversibility. Prospective studies involving pregnant and lactating females should be performed to assess the long-term effects of SSRIs on fetal and neonatal development. Molecular biology and genomics techniques should be used to conduct in-depth studies on the potential toxicity to aquatic organisms and the mechanisms of action of SSRIs to thoroughly study the mechanisms by which SSRIs affect germ cells, embryonic development, and reproductive system maturation. To comprehensively assess the risks and benefits of SSRI use, the severity of the disease, the drug dosage, and the duration of treatment should be considered in combination in patients with depressive disorders. Long-term ecological monitoring is necessary to study the cumulative effects and long-term impact of SSRIs and their metabolites in different ecosystems and their potential reproductive system toxicity. Finally, regulatory and protective measures must be explored to protect aquatic environments against drug contamination.

## 7 Summary and outlook

SSRIs are the most widely used antidepressant drugs in the world; however, their effects on the reproductive system of patients are often overlooked, especially their impact on germ cell cytogenesis, embryonic development, and reproductive system maturation. Despite some controversies, such as drug concentration and timing, inconsistent conclusions due to different definitional parameters, and insufficient research on the mechanism of influence, these findings can help clinicians choose the most appropriate treatment plan and clinical follow-up. Despite the proven efficacy of drugs that ameliorate the adverse effects of SSRIs, the small sample sizes of studies and the lack of deeper mechanistic elaboration have slowed the clinical application of these research findings. Larger randomized controlled trials as well as basic research studies are urgently needed to further explore the reproductive system pathogenesis induced by SSRIs and to identify better strategies for the use of SSRIs in treating diseases while also reducing their reproductive toxicity.
